# Gene therapy for colorectal cancer by an oncolytic adenovirus that targets loss of the insulin-like growth factor 2 imprinting system

**DOI:** 10.1186/1476-4598-11-86

**Published:** 2012-11-21

**Authors:** Zhen-Lin Nie, Yu-Qin Pan, Bang-Shun He, Ling Gu, Li-Ping Chen, Rui Li, Ye-Qiong Xu, Tian-Yi Gao, Guo-Qi Song, Andrew R Hoffman, Shu-Kui Wang, Ji-Fan Hu

**Affiliations:** 1 Central Laboratory, Nanjing First Hospital, Nanjing Medical University, Nanjing, 210006, Jiangsu Province, China; 2 Department of Life Sciences, Nanjing Normal University, Nanjing, 210046, Jiangsu Province, China; 3 Department of Medicine, Stanford University Medical School, Palo Alto, CA, 94304, USA; 4 Department of Medicine, PAIRE, VA Palo Alto Health Care System, Palo Alto, CA, 94304, USA

**Keywords:** Genomic imprinting, IGF2, Oncolytic adenovirus, Colorectal cancer, Targeted therapy

## Abstract

**Background:**

Colorectal cancer is one of the most common malignant tumors worldwide. Loss of imprinting (LOI) of the insulin-like growth factor 2 (IGF2) gene is an epigenetic abnormality observed in human colorectal neoplasms. Our aim was to investigate the feasibility of using the IGF2 imprinting system for targeted gene therapy of colorectal cancer.

**Results:**

We constructed a novel oncolytic adenovirus, Ad315-E1A, and a replication-deficient recombinant adenovirus, Ad315-EGFP, driven by the IGF2 imprinting system by inserting the H19 promoter, CCCTC binding factor, enhancer, human adenovirus early region 1A (E1A) and enhanced green fluorescent protein (EGFP) reporter gene into a pDC-315 shuttle plasmid. Cell lines with IGF2 LOI (HCT-8 and HT-29), which were infected with Ad315-EGFP, produced EGFP. However, no EGFP was produced in cell lines with maintenance of imprinting (HCT116 and GES-1). We found that Ad315-E1A significantly decreased cell viability and induced apoptosis only in LOI cell lines in vitro. In addition, mice bearing HCT-8-xenografted tumors, which received intratumoral administration of the oncolytic adenovirus, showed significantly reduced tumor growth and enhanced survival.

**Conclusions:**

Our recombinant oncolytic virus targeting the IGF2 LOI system inhibits LOI cell growth in vitro and in vivo, and provides a novel approach for targeted gene therapy.

## Background

Malignant neoplasms are responsible for nearly 7.5 million deaths representing 13% of all mortality and 5% of the global burden of disease in terms of disability-adjusted life years lost [1]. Colorectal cancer is the third most common cancer in men (10% of the total) and the second in women (9.4% of the total) worldwide [2]. Conventional chemotherapeutic agents, although often effective, are highly toxic because of their lack of selectivity for cancer cells. As a result, efforts have focused on developing interventions that target tumor-specific genes using techniques to construct tumor-selective replicating viruses.

Genomic imprinting is an epigenetic modification of a gene based on its parental origin, which results in monoallelic expression [3]. For most genes, both paternal and maternal alleles are expressed, but for some genes, only one allele is expressed, which is known as maintenance of imprinting (MOI). In contrast, loss of imprinting (LOI) is the reactivation of the silenced allele of an imprinted gene, leading to perturbation of monoallelic expression. LOI is closely related to the occurrence of malignant tumors [4]. The first endogenous imprinted gene identified was mouse insulin-like growth factor 2 (IGF2) encoding a critical fetal-specific growth factor that is only expressed from the allele inherited from the father [5]. IGF2 and H19 genes are reciprocally imprinted, and regulated by the enhancer, DNA differentially methylated domain (DMD), and promoter. The current model for the mechanism underlying this reciprocal imprinting proposes a critical role for CCCTC binding factor (CTCF) that binds to the unmethylated maternal DMD. Binding of target sequence elements by CTCF can block the interaction between enhancers and promoters, thereby limiting the activity of enhancers for certain functional domains. In recent years, researchers have elucidated the important roles of methylated regions and CTCF complexes that combine with the unmethylated maternal DMD of IGF2 imprinting [6-9]. Hu et al. [6] transferred nuclei from human tumor cells to show loss of IGF2 imprinting in enucleated mouse and human fibroblasts that had maintained normal IGF2 imprinting. After nuclear transfer, the abnormal tumor epigenotype was corrected by in vitro reprogramming, suggesting that LOI is associated with the loss of activity of non-CTCF trans-imprinting factor(s) that are either inactivated or mutated in tumors. In addition to blocking enhancers, CTCF can act as a chromatin barrier by preventing the spread of heterochromatin structures. In mice, it has been shown that CTCF serves as a strategic protein that implements DNA loops [7,8] and helps to silence DNA regions by binding and recruiting the polycomb repressive complex 2 [9]. Recently, Zhang et al. [10] extended the examination of the role of CTCF in orchestrating long-distance intrachromosomal looping in the human IGF2/H19 imprinting domain, and demonstrated that interruption of intrachromosomal looping by CTCF decoy proteins abrogates genomic imprinting of human IGF2. This observation confirmed that the inactivation or mutation of CTCF complexes is closely associated with the epigenetic IGF2 LOI in tumor cells. In our previous study, we found that loss of IGF2 imprinting is present in both colorectal tissues and cancer cell lines (HCT-8, HT-29, HCT15, and SW1222). No such phenomenon has been observed in normal tissue cells [6]. Furthermore, we successfully confirmed targeted tumor gene therapy based on IGF2 LOI [11].

Adenoviruses rapidly infect a broad range of human cells, including proliferating and quiescent cells, which tend to yield high levels of adenoviral particles [12]. In addition, adenoviral vectors are relatively easy to manipulate, purify and manufacture using recombinant DNA techniques, and transgenes generally do not undergo changes during successive rounds of viral replication. On the other hand, adenoviral vectors have poor targeting toward tumors and a low efficiency of gene transfer [13]. Abnormal biological characters shared by multiple cancers have enabled optimization of genetically engineered viruses that replicate exclusively in tumor cells (termed replication-selective adenoviral vectors), while not affecting normal cells. Adenovirus H101 has been clinically approved in China for the treatment of several malignancies. E1B and parts of the E3 regions have been deleted in this virus that cannot normally replicate and only lyses cells in which p53 is inactive. The cell cycle gatekeeper p53 is commonly observed to be inactive in tumors. Our present study includes four cell lines that have various IGF2 imprinting and p53 mutations (Table 1), and infection with H101 serves as the positive control.

**Table 1:** Analysis of p53 mutation and IGF2 gene imprinting in human tumor cells

**Cell lines**	**p53 status**	**IGF2 Imprinting**
GES-1	Wild type	MOI
HCT116	Wild type	MOI
HCT8	Wild type	LOI
HT29	Mutation	LOI

LOI: Loss of IGF2 imprinting; MOI: Maintenance of IGF2 imprinting.

In our previous study, a recombinant replication-defective adenovirus carrying the IGF2 imprinting system and the DT-A gene was successfully constructed, in which Ad-DT-A effectively kills tumor cells showing IGF2 LOI [11]. Replication-defective adenoviral vectors cannot continuously renew after initial activation, resulting in only transient expression of the DTA gene in LOI colorectal cancer cells.

One of the critical functions of the human adenovirus early region 1A (E1A) protein is to activate transcription of early viral genes [14]. This region activates viral gene expression by interacting with and recruiting cellular transcription machinery to the regulatory regions of early viral genes. Recent evidence indicates that the Ad5 E1A gene has a tumor suppressor role and exhibits a dual anti-tumor effect [15]. The E1A protein inhibits over-expression of erbB2 and protease genes, blocks NF-Kβ activity and then, as an antitumor effect, increases the expression of nm23 and p53 proteins [16]. Furthermore, E1A protein induces cell cycle progression from G0/G1 to S phase by binding and repressing the function of intracellular proteins such as pRb and p300, leading to cancer cell lysis by massive up-regulation of viral replication [17]. In this study, we constructed a novel replication-selective adenovirus based on the IGF2 LOI system to target colorectal cancers.

## Results

### Construction and characterization of the oncolytic adenovirus Ad315-E1A

**Figure 1:**
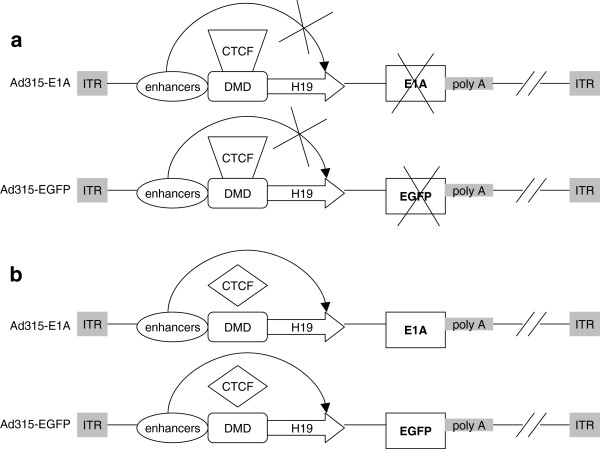
**Construction and expression characterization of Ad315-E1A and Ad315-EGFP in cell lines with different IGF2 imprinting status.** (**a**) In IGF2 MOI cell lines, CTCF binding factors are bound to DMD sites and the enhancer cannot control the H19 promoter, resulting in no expression of E1A or EGFP. (**b**) In IGF2 LOI cell lines, H19 has promoter activity that initiates expression of the downstream sequence due to CTCF complex regression. ITR: inverted terminal repeats.

The oncolytic adenovirus Ad315-E1A was successfully constructed in this study, which was regulated by the IGF2 imprinting system. A control virus, Ad315-EGFP, was also constructed (Figure 1). In MOI cells, the active CTCF complex can bind to the DMD, blocking the activity of the enhancer and inhibiting the expression of downstream genes, but the inactive CTCF complex in LOI cells cannot bind to the DMD, leading to increased expression of downstream genes as a result of promoter activation by the enhancers.

**Figure 2:**
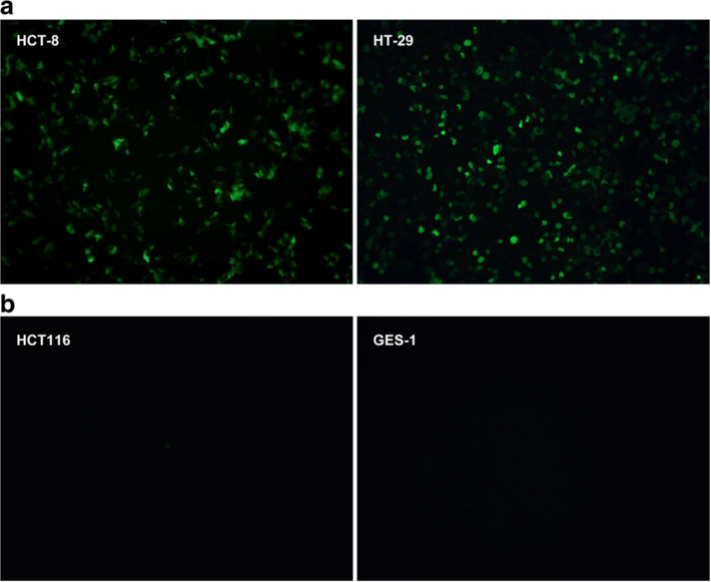
**Cell-specific expression of EGFP in four cell lines infected with recombinant adenoviral vectors.** (**a**) Positive expression of EGFP in LOI cell lines. Ad315-EGFP infection (10 PFU/cell) induced EGFP expression in HCT-8 and HT-29 cells. (**b**) Negative expression of EGFP in MOI cell lines. Fluorescence images show no EGFP expression in GES-1 cells and only weak EGFP expression in HCT116 cells infected with Ad315-EGFP (10 PFU/cell).

To test the applicability of the expression system, we used an enhanced green fluorescent protein (EGFP) reporter gene. After infection of cells with Ad315-EGFP (10 plaque-forming units [PFU]/cell) for 24 h, EGFP expression was observed in LOI cells lines HCT-8 and HT-29. However, in the HCT116 cell line, which maintains normal IGF2 imprinting, only weakly positive EGFP expression was observed (Figure 2). Thus, the viral gene therapy system only expressed the reporter gene in cells with abnormally maintained IGF2 imprinting.

**Figure 3:**
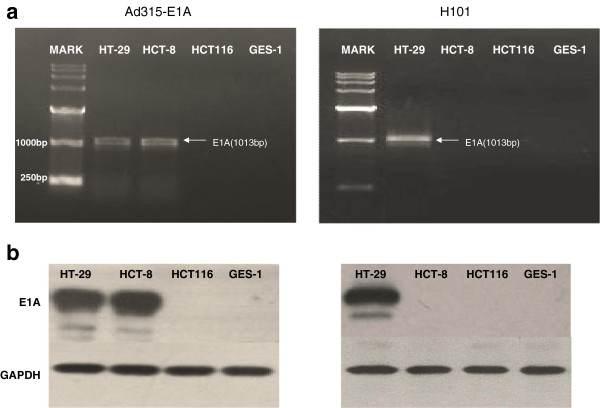
**E1A mRNA and protein expression in cells infected with adenoviral vectors carrying the E1A gene.** (**a**) RT-PCR analysis of E1A mRNA expression in four cell lines at 24 h after infection with Ad315-E1A or H101 (10 PFU/cell). (**b**) Western blot analysis of E1A protein expression in four cell lines at 48 h after infection with Ad315-E1A or H101 (10 PFU/cell). In the Ad315-E1A group, E1A mRNA and protein were expressed in HCT-8 and HT-29 cells, but not in GES-1 and HCT116 cells. E1A mRNA and protein expression was only detected in the P53 mutant cell line HT-29 in the H101 group.

We also examined E1A mRNA expression in four cancer cell lines (HCT-8, HT-29, HCT116, and GES-1) that were infected with Ad315-E1A and H101 (10 PFU/cell). Cells were harvested at 24 h after infection to determine E1A mRNA expression by RT-PCR, and E1A protein expression was determined by western blot at 48 h after infection. As shown in Figure 3, in the Ad315-E1A group, E1A mRNA and protein were expressed in LOI cell lines (HCT-8 and HT-29), but not in the MOI cell line (HCT116) and normal cell line (GES-1). H101 infection resulted in obvious E1A mRNA and protein expression in the p53 mutant cell line HT-29 compared with that in p53 wild-type cell lines (HCT116, HCT-8, and GES-1).

### Growth inhibition and cytotoxicity of LOI tumor cells by Ad315-E1A infection

**Figure 4:**
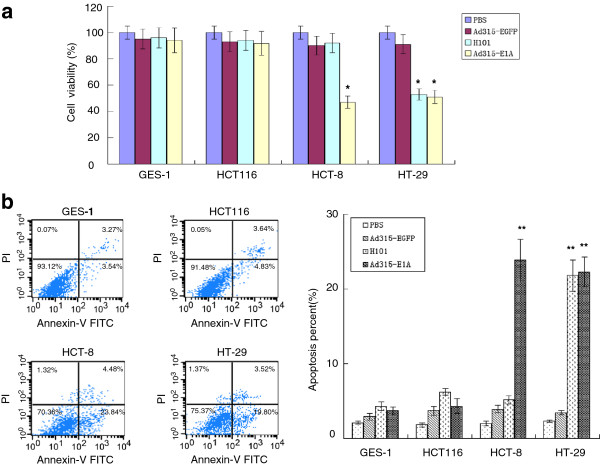
**In vitro effect of adenoviral vectors carrying the E1A gene.** (**a**) Cell viability was determined by an MTT assay at 72 h after infection with recombinant adenoviral vectors (10 PFU/cell). Graphs are representative of three separate experiments. (**b**) Apoptosis was investigated using flow cytometric analysis at 72 h after infection with Ad315-E1A, Ad315-EGFP or H101 (10 PFU/cell). The bottom right quadrants in dot plots show the apoptosis of various cell lines infected with Ad315-E1A. Data are representative of three separate experiments.

We were interested in examining whether Ad315-E1A treatment would effectively suppress tumor cell proliferation. For this purpose, the cytotoxic effects of the oncolytic adenovirus expressing the E1A gene were investigated in all cell lines. As shown in Figure 4a, the MOI cell line HCT116 and normal cell line GES-1, which were infected with Ad315-E1A, remained fully viable for 72 h after infection. However, the LOI cell lines (HCT-8 and HT-29) displayed decreased viability (p < 0.05). Growth inhibition was also improved compared with that of H101-infected cells with various p53 activities. The LOI-targeting oncolytic adenoviral vector Ad315-E1A induced cytopathic effects as efficiently as that of the oncolytic adenovirus H101 in the LOI and p53-inactive cell line (HT-29), whereas the growth inhibition ability of H101 was more strongly attenuated in the wild-type p53 and LOI cancer cell line (HCT-8) (p > 0.05).

We further examined apoptosis in all cell lines with varied IGF2 imprinting using flow cytometry at 72 h after infection. Ad315-EFGP infection served as a negative control to enable us to evaluate the cytopathic effect of adenoviral infection itself. As shown in Figure 4b, the percentages of cells undergoing apoptosis in LOI cell lines, HCT-8 and HT-29, were approximately 25% and 20%, respectively, which was higher than that in the control group at 3% (p < 0.01). In contrast, the percentage of apoptosis in HCT116 cells with normal IGF2 imprinting was not increased compared with that in the control group (p > 0.05). Moreover, the cytopathic effects of Ad315-E1A and H101 infections showed no significant difference in HT-29 colorectal carcinoma cells at the same multiplicity of infection (p > 0.05).

### Antitumor efficacy of Ad315-E1A in nude mice

**Figure 5:**
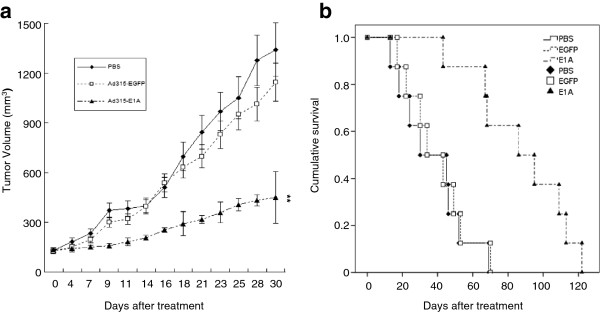
**Anti-tumor activity of Ad315-E1A in HCT-8 xenograft models.** (**a**) Average volume of subcutaneous tumors after receiving various treatments. Values represent the means ± SD for eight mice per group. Statistical differences were evaluated by one-way analysis of variance. **p < 0.01 vs. tumor volume of the Ad315-EGFP group. (**b**) Percentage of mouse survival analyzed by the Kaplan-Meier method.

Considering the potential of Ad315-E1A treatment in vitro, we investigated its antitumor efficacy in vivo. As shown in Figure 5a, tumor growth was slower in the Ad315-E1A-treated group than that in other groups. On day 30 after treatment initiation, the mean tumor volumes of phosphate-buffered saline (PBS)-, Ad315-EGFP- and Ad315-E1A-treated mice were 1342.4, 1145.0, and 451.8 mm^3^, respectively. Although no significant difference existed between PBS- and Ad315-EGFP-treated groups, significant antitumor efficacy was observed in the Ad315-E1A-treated group compared with that in other groups (p < 0.05). Moreover, the mean survival times for mice treated with PBS, Ad315-EGFP or Ad315-E1A were 37, 40 and 88 days, respectively. Ad315-EGFP treatment did not increase the survival time, compared with that of PBS treatment (p > 0.05), but Ad315-E1A treatment prolonged the survival time compared with that of treatment with PBS or Ad315-EGFP (p < 0.05, Figure 5b).

### E1A expression and apoptotic cells in tumors

**Figure 6:**
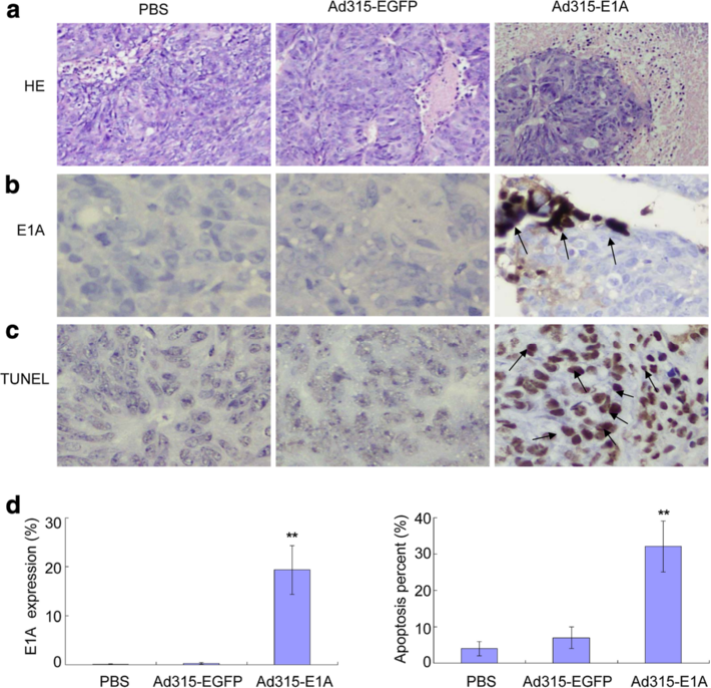
**Enhanced E1A expression and apoptosis in vivo by Ad315-E1A treatment.** (**a**) Histopathological responses in xenografted tumors on day 7 after treatment (H&E staining; original magnification: ×100). (**b**) E1A protein expression as measured by immunohistochemical staining. (**c**) Cell apoptosis in tumors after receiving the last treatment, as measured by a TUNEL assay. Original magnification: ×400. (**d**) Analysis of the E1A expression rate and apoptosis percentage.

Hematoxylin and eosin (H&E)-stained xenograft sections from Ad315-E1A-treated mice showed pathological signs of tumor necrosis obviously exceeding that of other groups (Figure 6a). To better understand the mechanism underlying the enhanced anti-tumor activity, we measured E1A protein expression and apoptosis in tumors. E1A protein was measured by immunohistochemical staining of tumor samples collected on day 7 after treatment (Figure 6b). E1A protein was observed at low levels in pathological sections from mice treated with Ad315-EGFP or PBS alone. However, in pathological sections infected by Ad315-E1A, E1A protein expression was markedly increased. The expression rates of E1A protein in HCT-8 cells treated with PBS, Ad315-EGFP or Ad315-E1A were 0.1 ± 0.1, 0.3 ± 0.2 and 20 ± 5%, respectively.

In agreement with E1A protein expression, terminal deoxynucleotide transferase-mediated dUTP nick-end labeling (TUNEL) assays revealed large numbers of apoptotic cells in tumors infected with Ad315-E1A (Figure 6c). In mice that received PBS or Ad315-EGFP, apoptotic cells were sparsely distributed in sections. The apoptotic indexes of HCT-8 tumor xenografts treated with PBS, Ad315-EGFP or Ad315 -E1A were 4 ± 2, 7 ± 3 and 32 ± 7%, respectively. Thus, the efficacy of tumor therapy was closely related to the enhanced apoptosis induced by Ad315-E1A infection.

## Discussion

In the present study, we successfully constructed a novel replication-selective adenovirus, Ad315-E1A, and a replication-deficient adenovirus, Ad315-EGFP. Our data show a significant effect of Ad315-E1A against tumor growth both in vitro and in vivo. This observation suggests that replication-selective adenoviruses driven by the IGF2 imprinting system may be used as a novel anti-cancer agent with a high therapeutic potential.

Using viruses as a genetic carrier offers an attractive alternative or promising integration with traditional therapeutic regimens for cancer treatment [18]. Among viral-based therapies and oncolytic agents, adenoviruses have emerged as a promising vector that is already being used for the treatment of solid tumors in humans [19]. Adenoviruses are non-enveloped viruses with linear double-stranded DNA, which infect cells by binding to the coxsackie and adenovirus receptor expressed on the surface of target cells [20]. As vectors for oncolytic therapies, these viruses have many advantages over other vectors, including the capability of transducing and replicating in dividing and non-dividing cells, their ease of manipulation, and a naturally lytic replication cycle [21-24]. All of these features highlight the utility of adenoviruses for in vitro production and in vivo curative effects [25]. Since 1993, more than 300 clinical trials based on adenoviral vectors have been performed [20] with promising outcomes. The first clinical results of trials based on adenoviruses as an oncolytic therapeutic agent have been promising, and show the clinical safety and feasibility of this approach [26]. In our study, we constructed an oncolytic adenoviral vector and demonstrated its effective killing of cancer cells both in vitro and in vivo.

IGF2 encodes the mitogenic anti-apoptotic peptide IGF-II that is overexpressed in several human cancers cells. In colorectal cancer, it has been reported that LOI of IGF2 occurs in 44% of informative colorectal cancers that are linked to microsatellite instability [27]. Additional studies have reported a range from 33% to 87% of sporadic colon cancers that were related to LOI of IGF2 [28,29].

Based on these findings, we theorized that we could build a kind of oncolytic adenovirus specific for replication in LOI tumor cells for colorectal cancer therapy. In this proof-of-concept study, we constructed adenoviral vectors, Ad315-E1A and Ad315-EGFP, which carry the three enhancer elements, DMD and promoter based on IGF2 LOI. Initially the utility of our expression system was tested using EGFP reporter assays. The results showed that green fluorescence was detected only in LOI cells. By increasing the multiplicity of infection and extending the time of infection, we found that the results were unchanged. To further demonstrate the selective efficacy of the LOI system, we used the oncolytic adenovirus H101, which has been clinically approved in China to treat malignancies, as a positive control for specific lysis of tumor cells by targeting the inactivated p53 in tumors. We used RT-PCR and western blotting to detect E1A expression at mRNA and protein levels, respectively, in Ad315-E1A-infected cells, and confirmed E1A expression in LOI colorectal cell lines. Furthermore, in H101-infected cells, E1A mRNA and protein was only detected in the p53 mutant cell line HT-29. All of the above results indicate that the replication-selective system that we used is practicable and effective.

In this study, the therapeutic potential of Ad315-E1A was tested by an MTT assay and flow cytometry, and the results clearly support the rationale of our hypothesis, in which Ad315-E1A effectively suppressed the growth of tumor cells and induced obvious cytotoxicity in LOI tumor cells. Therefore, we compared the therapeutic effect between IGF2 regulatory sequences and H101 for colorectal cancer treatment. The data showed that there was no detectable cytotoxicity in HCT-116 and GES-1 cells infected with Ad315-E1A or H101 (10 PFU/cell), whereas Ad315-E1A infection achieved significant growth inhibition of the IGF2 LOI and p53-active cell line (HCT-8) relative to that by H101 infection. In addition, both oncolytic viruses exhibited superior efficiency to inhibit growth of the HT-29 (p53 mutant and LOI) cell line. These results suggest that the IGF2 LOI system in our viral vector enabled efficient adenovirus replication in host cells using the E1A endogenous promoter.

Using the human colorectal cancer cell line HCT-8 as a model, we showed that this therapeutic strategy significantly increased cancer cell death and apoptosis caused by virus replication in vivo. Despite Ad315-E1A treatment significantly inhibiting tumor growth and prolonging the survival of tumor-bearing mice, the tumors were not eliminated completely. Previous research also found that oncolytic viruses have a limited potential to eradicate tumors when used as a monotherapy [30-32]. Thus, oncolytic viruses are often used in combination with other modalities, such as chemotherapy [33], radiotherapy [34], or arming oncolytic adenoviruses with therapeutic genes [35], to improve the anti-tumor efficacy. In our next study, we will investigate appropriate methods to enhance the potency of the novel recombinant adenovirus for cancer gene therapy.

## Conclusions

In summary, our results show that an oncolytic adenovirus regulated by the IGF2 LOI system confers a significant anti-tumor effect by induction of apoptosis in vitro and in vivo in human colorectal cancer cells. LOI exists in a wide variety of tumors, and although more investigations are required for future use, our preliminary data support the use of oncolytic viruses in the context of the IGF2 LOI system as a novel approach for cancer therapy.

## Methods

### Cell lines and culture

HCT-8, HCT116, HT-29 (human colon cancer cell lines) and GES-1 (normal human cell line) were obtained from the Shanghai Cell Collection, Chinese Academy of Sciences. The HEK293 cell line (human embryonic kidney cells containing the E1A region of the adenovirus) was obtained from Microbix Inc. (Ontario, Canada). HEK293, GES-1, and HCT-8 cell lines were maintained in Dulbecco’s modified Eagle’s medium (DMEM; Hyclone, UT, USA) supplemented with 10% fetal bovine serum (FBS; Hyclone). HCT116 and HT-29 cells were maintained in RPMI 1640 (Invitrogen, CA, USA) supplemented with 10% FBS. Cells were maintained in a humidified incubator at 37°C with 5% CO_2_.

### Plasmid construction and incorporation into adenoviral vectors

The original adenoviral shuttle plasmid that we used in this study was pDC315, and AD5 PBHGLOX1, 3CRE (Microbix Biosystems, Ontario, Canada). Mouse H19 enhancer exons 1 (258 bp) and 2 (360 bp) were amplified by PCR from mouse genomic DNA and then linked as a single fragment by PCR. The enhancer was cloned into the pDC315 plasmid using restriction endonucleases Xbal and EcoRI. Subsequently, mouse DMD exons 1–2 (429 bp), 3 (207 bp) and 4 (156 bp) were also amplified by PCR from genomic mouse DNA and then linked as a single fragment by PCR. The DMD was cloned downstream of the enhancer using restriction endonucleases EcoRI and NheI. The mouse H19 promoter (302 bp) was amplified by PCR from mouse genomic DNA using the following primers: forward, 5’ GCGCTAGCCCACCGTTCTATGAAGGGCTTC 3’ (containing a NheI site) and reverse, 5’ AAGGATCCTCATCAGCGCCCATCTCTAGCC 3’ (containing a BamHI site), and then cloned downstream of the DMD using restriction endonucleases NheI and BamHI. The human adenovirus E1A sequence (1013 bp) was amplified by PCR from a TOP-K plasmid, which was kindly provided by Dr. Ji-Fan Hu (Stanford University Medical School), using the following primers: forward, 5’ CCCGGATCCGGGCCCTATGAGACATATTATCT 3’ (containing a BamHI site) and reverse, CGCGTCGACCGCAATCACAGGTTTACACCTTA 3’ (containing a SalI site).

The EGFP reporter gene from the pEGFP-C1 vector (Clontech, Mountain View, CA, USA) and the E1A gene were then inserted downstream of the H19 promoter using restriction endonucleases BamHI and SalI to construct pDC315-enhancer-DMD-H19-EGFP and pDC315-enhancer-DMD-H19-E1A. Inserts were confirmed by DNA sequencing. The adenovirus Ad315-E1A was constructed by homologous recombination techniques using pDC315-enhancer-DMD-H19-E1A and the adenovirus packaging plasmid PBHGLOX1, 3CRE in HEK293 cells with Lipofectamine 2000 (Invitrogen Life Technologies, CA, USA). A standard replication-deficient adenovirus, Ad315-EGFP, was constructed by cotransfection of the adenovirus shuttle vector containing EGFP with an E1A/B-deleted adenoviral backbone vector.

Adenoviruses were plaque purified, propagated in HEK293 cells, and purified again by a CsCl gradient according to standard techniques. Functional particle titers of all adenoviruses were determined by a plaque assay using HEK293 cells. The positive control adenovirus H101 was kindly provided by Dr. Sheng-Fang Ge (Shanghai Jiao Tong University School of Medicine).

### Virus infection

Cells were seeded in 96-well plates at a density of 10,000 cells/well for a 3-(4, 5-dimethylthiazol-2-yl)-2, 5-diphenyl tetrazolium bromide (MTT) assay, or in 6-well plates at a density of 1000,000 cells/well for RT-PCR and flow cytometric analysis. Then, cells were incubated with various concentrations of Ad315-EGFP, Ad315-E1A, and H101 in serum-free DMEM at 37°C for 90 min. After incubation, serum-free DMEM containing the viruses was replaced with normal growth medium. The infected cells were maintained at 37°C until use in assays.

### EGFP and E1A expression analyses

EGFP expression was examined at 24 h after infection with adenoviral vectors (10 PFU/cell) under an Axioskop 2 microscope (Carl Zeiss, Oberkochen, Germany) with a fluorescent filter set (excitation 450–490 nm). E1A mRNA expression was determined by RT-PCR. Total RNA was extracted using Trizol (Invitrogen Life Technologies) according to the manufacturer’s instructions. First strand cDNA synthesis was performed in a total volume of 25 μl containing 2 μg RNA, 0.5 μg primer and 200 U M-MLV reverse transcriptase. cDNA was subsequently amplified in 50 μl reaction volumes containing 0.4 μmol/l of each E1A primer and 1.25 U Taq DNA polymerase (TaKaRa, Dalian, China). The amplification conditions were pre-denaturation at 94°C for 5 min, followed by 35 cycles of 94°C for 40 s, 60°C for 40 s and 72°C for 60 s, and a final extension of 72°C for 7 min. PCR products were electrophoresed on a 1% agarose gel containing ethidium bromide and then visualized under UV light.

### Western blotting to detect E1A protein expression

Cells were harvested and lysed by three cycles of freeze/thawing at −80°C. Cell lysates were separated by 12% sodium dodecyl sulfate polyacrylamide gel electrophoresis and then transferred onto nitrocellulose membranes (Amersham Pharmacia Biotech AB, Uppsala, Sweden). Membranes were probed with a mouse monoclonal antibody against E1A (Abcam, Boston, MA, USA) and then a horseradish peroxidase-conjugated goat anti-mouse IgG. Proteins were visualized using Lumi-Light Western Blotting Substrate (Roche Molecular Biochemicals).

### Cytotoxicity assay

Cytotoxicity was assessed using an MTT assay that was performed as described previously using a Cell Counting Kit-8 (CCK-8; Dojindo Laboratories, Japan) [36]. Briefly, cells were seeded in 96-well plates at a density of 10,000 cells/well. Then, cells were infected with recombinant adenoviral vectors (10 PFU/cell). Cell growth and viability were assayed at 72 h after incubation with CCK-8 kit reagents by measuring absorbances at 450 nm in a microplate reader (Bio-Rad, Richmond, CA, USA). Each sample was assayed in quadruplicate, and experiments were repeated at least twice.

### Apoptosis assay

Quantitative evaluation of apoptosis was performed using flow cytometry after double staining with an annexin V-fluorescein isothiocyanate apoptosis detection kit to discrimination between early apoptotic (annexin V-positive) and necrotic ( annexin V/propidium iodide double-positive) cells. The cells (100,000 cells/well) were cultured in 6-well plates, and then infected with Ad315-E1A at 10 PFU/cell. Cell apoptosis was analyzed at 72 h after infection with Ad315-EFGP (10 PFU/cell) serving as a negative control.

### Tumor xenografts

Tumor xenografts were established by subcutaneous injection of 5 × 10^6^ HCT-8 cells into the right flank of 4–6-week-old female athymic nude mice, which was approved by the Experimental Animal Center of University of Yangzhou, Yangzhou, China. The average tumor volume was measured by the formula: volume = (length × width^2^) / 0.5. When tumors reached approximately 100 mm^3^, xenografted mice were randomly divided into three treatment groups with eight mice in each group. The Ad315-E1A group received intratumoral injections of 1 × 10^8^ Ad315-E1A viral particles three times every other day. Tumors injected with Ad315-EGFP viral particles at the same dosage served as a viral vector control, and those injected with PBS served as a negative control. The tumor size was measured by vernier calipers every 3 days. Mice were then euthanized by cervical dislocation at a predetermined interval of observation. Tumors were dissected out and stored in 40% formalin. For histological analysis, fixed tumors were embedded in OCT compound and then cut into 5–7 mm sections using a Cryocut microtome (Leica, Germany). Mouse survival was recorded in a separate experiment. Animal experiments were performed in accordance with institutional guidelines for animal care by Nanjing Medical University.

### Immunohistochemistry

To immunohistochemically stain the E1A protein, the tumor and neighboring tissues were fixed in 10% formalin and embedded in paraffin for staining. Tumor or tissue sections were incubated at 4°C overnight with a mouse anti-human E1A antibody (Abcam, Boston, MA, USA) at a dilution of 1:50. Sections were rinsed in PBS-T (0.05% Triton X-100 in PBS), followed by incubation with a goat anti-mouse secondary antibody at a 1:500 dilution for 1 h at room temperature. Sections were subsequently incubated with streptavidin-horseradish peroxidase (BD Biosciences) and diaminobenzidine substrate to develop the colorimetric reaction. The number of E1A-positive cells was counted in five random fields at ×400 magnification under a light microscope and averaged. Only cells with distinct staining were counted. The positivity rate was used to grade the expression levels.

### TUNEL assay

Apoptosis of tumor cells was detected using a TUNEL assay performed with an In Situ Cell Death Detection Kit (Roche, Mannheim, Germany) following the manufacturer’s instructions. To stain apoptotic cells, tumor samples were fixed with 10% formaldehyde and paraffin-embedded sections were prepared. The number of TUNEL-positive cells was counted in five random fields at ×400 magnification under a light microscope, and the apoptosis index for each field was calculated as the percentage of TUNEL-positive cells relative to the total number of cells.

### Statistical analysis

Experimental data were presented as the mean ± standard deviation (SD) and assessed using the Student’s *t*-test and one-way analysis of variance. Differences among the results of in vivo survival experiments were assessed by the Kaplan-Meier method. Results were considered statistically significant at p < 0.05.

## Competing interests

The authors declare that they have no competing interests.

## Authors’ contributions

SW, YP, and ZN designed the study and wrote the protocols. ZN performed most of the experiments. BH, LG, and LC contributed to administrative, technical or material support. RL, YX, and TG managed the literature searches and analyses. GS undertook the statistical analysis. ZN drafted the manuscript. AH and JH provided critiques of the manuscript. All authors read and approved the final manuscript.
